# New opioid use and risk of opioid-related adverse events among adults with intellectual and developmental disabilities in Ontario, Canada

**DOI:** 10.1192/bjo.2022.612

**Published:** 2022-11-28

**Authors:** Qi Guan, Siyu Men, Yona Lunsky, David N. Juurlink, Susan E. Bronskill, Hannah Wunsch, Tara Gomes

**Affiliations:** Institute of Health Policy, Management and Evaluation, University of Toronto, Canada; ICES, Toronto, Canada; and Li Ka Shing Knowledge Institute, St Michael's Hospital, Toronto, Canada; ICES, Toronto, Canada; ICES, Toronto, Canada; Department of Psychiatry, University of Toronto, Canada; and Azrieli Adult Neurodevelopmental Centre, Centre for Addiction and Mental Health, Toronto, Canada; Institute of Health Policy, Management and Evaluation, University of Toronto, Canada; ICES, Toronto, Canada; Department of Medicine, University of Toronto, Canada; and Sunnybrook Research Institute, Toronto, Canada; Institute of Health Policy, Management and Evaluation, University of Toronto, Canada; ICES, Toronto, Canada; and Sunnybrook Research Institute, Toronto, Canada; Institute of Health Policy, Management and Evaluation, University of Toronto, Canada; ICES, Toronto, Canada; Department of Critical Care Medicine, Sunnybrook Health Sciences Centre, Toronto, Canada; Department of Anesthesiology and Pain Medicine, University of Toronto, Canada; and Interdepartmental Division of Critical Care Medicine, University of Toronto, Canada; Institute of Health Policy, Management and Evaluation, University of Toronto, Canada; ICES, Toronto, Canada; Li Ka Shing Knowledge Institute, St Michael's Hospital, Toronto, Canada; and Leslie Dan Faculty of Pharmacy, University of Toronto, Canada

**Keywords:** Drug interactions and side-effects, drugs of dependence disorders, epidemiology, intellectual disability, opiate disorders

## Abstract

**Background:**

Individuals with intellectual and developmental disability (IDD) can have a high prevalence of pain, which can be managed with prescription opioids. However, the prevalence of substance use disorder is also high in this population, raising concern about opioid-related adverse events.

**Aims:**

To assess the risk of opioid-related adverse events following opioid initiation among adults with versus without IDD.

**Method:**

We conducted a population-based, propensity score matched cohort study on all adults starting prescription opioid therapy in Ontario, Canada, between January 2013 and December 2018. The outcomes of interest were opioid toxicity, new opioid use disorder (OUD) diagnosis and dose escalation (≥90 mg morphine or equivalent) in the year after opioid initiation. We used Cox proportional hazards models to assess the association between IDD diagnosis and each outcome.

**Results:**

The hazards of opioid toxicity and OUD were significantly higher in those with IDD compared with those without IDD in unmatched analyses (opioid toxicity hazard ratio 3.19, 95% CI 2.81–5.18; OUD hazard ratio 2.36, 95% CI 2.10–2.65), whereas the hazard of dose escalation was significantly lower (hazard ratio 0.76, 95% CI 0.66–0.88). Findings were no longer significant in propensity score matched models for opioid toxicity and dose escalation, whereas the hazard of OUD diagnosis was attenuated substantially in those with IDD (hazard ratio 0.79, 95% CI 0.68–0.91).

**Conclusions:**

IDD diagnosis is not a driver of opioid-related harm. The increased risk we observed is likely driven by various risk factors often present in this population.

Individuals with intellectual and developmental disabilities (IDD) are medically complex and can experience excess pain resulting from their disability as well as the medical tests and procedures conducted to manage their comorbidities.^1^ A recent estimate suggests a 70% prevalence of chronic pain among those with IDD,^2^ which is substantially higher than that of individuals without IDD (approximately 28%).^3^ Drug therapy, often using prescription opioids, is an option to manage pain in individuals with IDD in addition to physical therapy and psycho-behavioural treatment;^1^ however, the need for adequate pain management using opioids must be balanced against the known risks of overdose and development of substance use disorders following long-term use of these medications.^4,5^

Such concerns are particularly pronounced among individuals with IDD because this is a population with a higher prevalence of alcohol and substance use disorders than those without IDD, despite less widespread substance use.^6–8^ The number of individuals with IDD now living independently in community settings has increased dramatically,^9^ leading to benefits such as enhanced autonomy and social inclusion.^6^ However, these settings can also increase exposure to substance use,^10^ which may lead to harms given that individuals with IDD often exhibit personality dimensions associated with an increased risk of substance use disorder (i.e. impulsivity, negative thinking, anxiety sensitivity and sensation-seeking).^8^ Furthermore, individuals with IDD often manage a complex regimen of medications, which intrinsically may increase the risk of accidental toxicity owing to medication error or drug interactions.^11,12^ Although medication management supports like blister packs are available to support safe medication use among those with IDD, opioids are typically not provided in this form because of their ‘as-needed’ manner of use. Given the elevated risk of substance use disorder and the complex medical profiles of individuals with IDD, compounded with the high risk of potential adverse events associated with opioids,^4,13–15^ opioid therapy may be particularly risky in this population if therapy is not carefully managed.

## Aims

Despite anticipated high rates of opioid use to manage pain and elevated risks of substance use disorders among people with IDD, there is limited evidence examining prescription opioid use in this population. We sought to describe characteristics of new prescription opioid use among individuals with IDD and to examine the subsequent risks of opioid toxicity, opioid use disorder (OUD) and dose escalation, compared with individuals without IDD.

## Method

### Setting

We used a population-based, retrospective cohort design to study individuals (with and without IDD) in Ontario, Canada who started prescription opioid treatment between 1 January 2013 and 31 December 2018. As of 2021, Ontario was the most populous province in Canada, with almost 15 million residents^16^ who all have access to universal coverage for hospital and physician services. Data used in this study are held in databases at ICES^17^ (previously the Institute for Clinical Evaluative Sciences) in Toronto, Canada (www.ices.on.ca). These data-sets were linked with unique encoded identifiers and analysed at ICES. The main data-set used was the Narcotics Monitoring System database, which holds records of all controlled substances dispensed in community pharmacies in Ontario (i.e. opioids, barbiturates, benzodiazepines and stimulants). We used this to obtain details of all prescription opioids dispensed to our cohort. Further details on the data-sets used in this study are described in the Supplementary Materials available at https://doi.org/10.1192/bjo.2022.612. Use of these data was authorised under section 45 of the Ontario Personal Health Information Protection Act, which does not require review by a research ethics board.

### Study cohort

Our cohort consisted of adults (≥18 years of age) in Ontario who started prescription opioids between 1 January 2013 and 31 December 2018. The date of the first opioid dispensed was identified as the index date. New opioid therapy was defined as having no opioid prescriptions dispensed in the 6 months before index date. We excluded those who had a record of opioid toxicity or OUD in the 3 years before index date, to restrict to those without recent evidence of an OUD. We also excluded individuals who started opioid therapy at a daily dose of ≥90 mg morphine or equivalent (MEQ) because this dose should rarely be used at initiation and is therefore indicative of a patient who is not opioid naïve.^18^ We used a method developed by the Ontario Drug Policy Research Network to calculate daily opioid dose by converting opioid doses to milligrams of morphine equivalents, as described elsewhere.^19^ Since opioid use in palliative settings is considerably different from opioid use for acute and chronic pain, we also excluded those who were receiving palliative care in the 6 months before index date (see Supplementary Table 1 for palliative care and opioid toxicity definitions).

### Exposure

We stratified the cohort based on a history of an IDD diagnosis at index date, using a health claims algorithm developed by the Health Care Access Research and Developmental Disabilities Program.^20^ Individuals were identified as having IDD if they had any of the following three combinations of IDD-related healthcare interactions at any time before or on index date: one or more hospital visits (using the Canadian Institute for Health Information (CIHI) Discharge Abstract Database, CIHI-Same Day Surgery or Ontario Mental Health Reporting System databases) with an IDD diagnosis code; one or more emergency department visits (using the CIHI-National Ambulatory Care Reporting System database) with an IDD diagnosis code; or two or more out-patient physician visits (using the Ontario Health Insurance Plan (OHIP) database) with an IDD diagnosis code (Supplementary Tables 2 and 3). If none of these criteria are met, an individual would be classified as someone without IDD.

To minimise potential differences between the two exposure groups, we matched those with IDD to those without IDD, using a propensity score calculated by fitting a logistic regression model that included the following covariates: gender; age; rurality; Aggregated Diagnosis Groups (ADG) score from the Johns Hopkins ACG System version 10.0 for Windows (Johns Hopkins University, Maryland, USA, https://www.hopkinsacg.org/); number of in-patient, out-patient and ambulatory visits for any reason in the year before index date; number of psychiatric comorbidities; number of visits to a physician for mental health issues (in-patient or out-patient) in the year before index date; history of substance use disorder and previous use of stimulants or benzodiazepines. Each new opioid recipient diagnosed with IDD was then greedy matched with up to four new opioid recipients without an IDD diagnosis, according to gender, age (±1 year), logit of the propensity score (±0.2 s.d.), index date (±30 days), initial daily dose (±10 MEQ) and duration of action of the initial opioid (long-acting or immediate release). In a sensitivity analysis, we calculated a high-dimensional propensity score (HDPS) for each individual in the cohort and greedy matched each individual with IDD with up to four individuals without IDD by using the same variables described above, but replaced the logit of the propensity score with the logit of the HDPS.^21^ Additional details on the HDPS calculation are provided in the Supplementary Materials.

### Outcomes

The primary outcome was an opioid toxicity event (fatal or non-fatal), defined as a visit to the emergency department or an in-patient admission for opioid-related toxicity (ICD-10^22^ codes: T40.0, T40.1, T40.2, T40.3, T40.6) or an opioid-related death within 1 year following the index date. We censored individuals if they experienced a non-opioid-related death, hospital stay of >30 days or at the end of the observation window (1 year after index). Two secondary outcomes were defined as a new OUD diagnosis and dose escalation. New OUD diagnosis during the follow-up period was defined as the first occurrence of an emergency department, hospital or out-patient physician diagnosis code (ICD-10 code: F11; DSM-5^23^ codes: 304.00, 305.50; OHIP fee codes: K682, K682, K684) or any records of opioid agonist therapy. This outcome was censored on all-cause death and end of the study period. Dose escalation was defined as being dispensed a prescription with a daily dose of ≥90 MEQ during the observation window (all individuals received initial daily doses of <90 MEQ). This threshold was selected because it has been associated with an increased risk of adverse events, including motor vehicle collisions, increased emergency department visits and opioid-related death.^13,24–26^ For this outcome, observations were censored on all-cause death, 30-day hospital stay, opioid discontinuation (defined as having received no prescription opioid dispensed within the longer of 30 days or a period equal to twice the treatment duration of the previous prescription) or end of observation window.

### Statistical analyses

We used descriptive statistics to summarise baseline and opioid therapy characteristics of both the unmatched and matched cohorts. We compared the exposure groups by using standardised differences where a value >0.10 was deemed clinically meaningful.^27^ We used Cox proportional hazards models with robust variance estimators to estimate hazard ratios and 95% confidence intervals for each of the study outcomes in each cohort (unmatched, propensity matched and HDPS matched). We tested the proportional hazards assumption for all models by examining the plot of the log-negative-log survival function estimates versus the log of survival time, and by including an interaction between IDD diagnosis and time.

We conducted two exploratory analyses to further understand factors associated with opioid toxicity in our cohort. In the first analysis, we fit a multivariable cox model for the entire unmatched cohort to identify statistically significant correlates of opioid toxicity. In the second analysis, we replicated the unmatched, propensity score matched and HDPS matched cox regression models for our primary outcome with an additional censoring criterion of opioid discontinuation. This allowed us to determine the hazard of on-treatment opioid toxicity during the year after treatment initiation. All analyses were conducted with SAS Enterprise Guide version 7.15 for Windows (SAS Institute, North Carolina, USA).

## Results

This study included 3 951 779 individuals who started prescription opioid therapy during the 6-year study period, among whom 20 684 (0.5%) had IDD. We successfully matched 19 814 (95.8%) of adults with IDD to up to four individuals without IDD, resulting in a final cohort size of 97 522. Before matching, clinical and demographic characteristics differed considerably according to IDD diagnosis (Table 1). Specifically, individuals with IDD were younger (mean age 37 *v*. 50 years; standardised difference 0.70), more likely to be male (57.7 *v*. 46.5%; standardised difference 0.23) and tended to live in areas of lower income (29.3 *v*. 19.5% lowest income quintile; standardised difference 0.23) when compared with individuals without IDD. The IDD group also had a higher level of comorbidity (mean ADG score ± s.d.: 6.7 ± 3.9 *v*. 6.0 ± 3.4; standardised difference 0.19) and were more likely to have a history of alcohol and substance use disorders (3.4 *v*. 1.3%; standardised difference 0.14). After propensity score matching, exposure groups were more comparable, although some differences remained in income quintile of residence, prior health services utilisation and comorbidity profiles (Table 1). After HDPS matching, balance was improved further, with only differences in previous mental health hospital admission remaining (Supplementary Table 4).

**Table 1:** Cohort characteristics stratified by intellectual and developmental disability diagnosis, before and after propensity score matching

	Unmatched cohort		Propensity score matched cohort	
	IDD	No IDD	IDD	No IDD
	*N* = 20 684	*N* = 3 931 095	*N* = 19 814	*N* = 76 708
Demographics				
Age, mean ± s.d.	37.2 ± 17.0	49.7 ± 18.7^a^	37.1 ± 17.0	7.1 ± 17.0
Male, *n* (%)	11 943 (57.7)	1 829 924 (46.5)^a^	11 452 (57.8)	44 337 (57.8)
Income quintile, *n* (%)				
1	6053 (29.3)	764 644 (19.5)^a^	5733 (28.9)	15 955 (20.8)^a^
2	4457 (21.5)	784 699 (20.0)	4279 (21.6)	15 111 (19.7)
3	3592 (17.4)	789 542 (20.1)	3463 (17.5)	14 958 (19.5)
4	3328 (16.1)	796 431 (20.3)^a^	3200 (16.2)	15 188 (19.8)
5	3225 (15.6)	790 458 (20.1)^a^	3111 (15.7)	15 264 (19.9)^a^
Rural residence	2549 (12.3)	437 599 (11.1)	2448 (12.4)	9589 (12.5)
Health services utilisation in the year before index date, mean ± s.d.				
Number of emergency department visits	1.7 ± 4.2	0.7 ± 1.3^a^	1.3 ± 2.4	1.1 ± 2.0
Number of physician office visits (any reason)	7.9 ± 8.4	7.2 ± 7.0	7.5 ± 7.6	7.1 ± 7.5
Number of physician office visits (mental health)	1.8 ± 4.8	0.5 ± 2.4^a^	1.5 ± 3.8	1.3 ± 4.3
Previous hospital admission, *n* (%)	2467 (11.9)	304 221 (7.7)^a^	2113 (10.7)	6750 (8.8)
Previous mental health hospital admission, *n* (%)	1222 (5.9)	12 925 (0.3)^a^	821 (4.1)	1380 (1.8)^a^
Comorbidities and medication use, *n* (%)				
ADG score, mean ± s.d.	6.7 ± 3.9	6.0 ± 3.4^a^	6.5 ± 3.7	6.4 ± 3.6
Alcohol and substance use disorder	712 (3.4)	51 256 (1.3)^a^	594 (3.0)	1918 (2.5)
Cancer	1028 (5.0)	353 406 (9.0)^a^	979 (4.9)	3835 (5.0)
COPD	1649 (8.0)	384 844 (9.8)	1521 (7.7)	4526 (5.9)
Diabetes	2628 (12.7)	559 363 (14.2)	2450 (12.4)	6674 (8.7)^a^
Hypertension	3828 (18.5)	1 255 805 (31.9)^a^	3628 (18.3)	13 731 (17.9)
Rheumatoid arthritis	146 (0.7)	53 192 (1.4)	135 (0.7)	614 (0.8)
Benzodiazepine use (6 months before index date)	4377 (21.2)	299 370 (7.6)^a^	3728 (18.8)	15 342 (20.0)
Stimulant use (6 months before index date)	1161 (5.6)	28 130 (0.7)^a^	999 (5.0)	4066 (5.3)

IDD, intellectual and developmental disability; ADG, Aggregated Diagnosis Group; COPD, chronic obstructive pulmonary disease.

a. Meaningful difference based on standardised difference >0.10 when compared with the IDD group.

In general, characteristics of the initial opioid were similar between exposure groups before matching. Among those with IDD, there was a slightly higher percentage prescribed codeine combination products (63.5 *v*. 57.6%; standardised difference 0.12), a lower percentage prescribed less common opioids such as tramadol and buprenorphine for pain (6.3 *v*. 9.5%; standardised difference 0.12), a lower percentage with initial prescription durations longer than a week (15.4 *v*. 20.3%; standardised difference 0.13), and a higher percentage with concurrent benzodiazepine (15.3 *v*. 5.2%; standardised difference 0.34) and stimulant (4.3 *v*. 0.5%; standardised difference 0.25) use. After matching, all index opioid prescription characteristics were well-balanced between groups, except use of less common (‘other’) opioids (Table 2, Supplementary Table 5).

**Table 2:** Medication characteristics on index date and during observation window (365 days after opioid therapy initiation), before and after propensity score matching

	Unmatched cohort		Propensity score matched cohort	
	IDD	No IDD	IDD	No IDD
	*n* = 20 684	*n* = 3 931 095	*n* = 19 814	*n* = 76 708
Index opioid characteristics, *n* (%)				
Single opioid prescription on index date	20 479 (99.0)	3 891 330 (99.0)	19 654 (99.2)	76 171 (99.3)
Opioid type				
Codeine	495 (2.4)	70 861 (1.8)	472 (2.4)	1227 (1.6)
Codeine combination	13 143 (63.5)	2 265 336 (57.6)^a^	12 690 (64.1)	46 638 (60.8)
Fentanyl	≤5	516 (0)	≤5	≤5
Hydromorphone	1594 (7.7)	313 666 (8.0)	1464 (7.4)	4833 (6.3)
Meperidine	46 (0.2)	9605 (0.2)	43 (0.2)	230 (0.3)
Methadone (for pain)	0	≤5	0	0
Morphine	760 (3.7)	112 757 (2.9)	720 (3.6)	1994 (2.6)
Oxycodone	237 (1.1)	80 473 (2.0)	219 (1.1)	1151 (1.5)
Oxycodone combination	3211 (15.5)	723 388 (18.4)	3055 (15.4)	13 961 (18.2)
Other opioids	1304 (6.3)	374 355 (9.5)^a^	1,246 (6.3)	7,057 (9.2)^a^
Long-acting opioid	160 (0.8)	35 074 (0.9)	73 (0.4)	307 (0.4)
Daily dose >50 MEQ	3131 (15.1)	654 164 (16.6)	2929 (14.8)	10 816 (14.1)
Prescription duration >7 days	3184 (15.4)	796 832 (20.3)^a^	3006 (15.2)	12 810 (16.7)
Concurrent benzodiazepine prescription	3166 (15.3)	204,046 (5.2)^a^	2709 (13.7)	7978 (10.4)
Concurrent stimulant prescription	899 (4.3)	20 358 (0.5)^a^	773 (3.9)	2761 (3.6)
Observation window medication characteristics, *n* (%)				
Additional opioid prescription dispensed	6043 (29.2)	1 264 823 (32.2)	5636 (28.4)	23 396 (30.5)
Long-acting opioid during observation window	388 (1.9)	93 112 (2.4)	317 (1.6)	1381 (1.8)
Number of additional opioid prescriptions, mean ± s.d.	1.2 ± 5.7	0.9 ± 3.1	1.1 ± 5.2	0.9 ± 3.0
Opioid discontinuation	20 462 (98.9)	3 901 431 (99.2)	19 624 (99.0)	76 248 (99.4)

IDD, intellectual and developmental disability; MEQ, milligrams of morphine or equivalent.

a. Meaningful difference based on standardised difference >0.10 when compared with the IDD group.

In the unmatched analysis, IDD diagnosis was associated with a significantly higher hazard of opioid toxicity (2.10 *v*. 0.54 per 1000 person-years; hazard ratio 3.19, 95% CI 2.81–5.18) compared with no IDD diagnosis (Table 3). This association was no longer statistically significant after propensity score matching (hazard ratio 0.96, 95% CI 0.63–1.47) and HDPS matching (hazard ratio 1.07, 95% CI 0.70–1.65).

**Table 3:** Hazard of opioid toxicity, new opioid use disorder diagnosis and dose escalation opioids during the year after initiating opioid therapy, before and after propensity score matching

	Total number of individuals	Total number of person-years of follow-up	Number of events	Incidence rate per 1000 person-years (95% CI)	Hazard ratio (95% CI)
Unmatched cohort					
Opioid toxicity					
No IDD diagnosis	3 931 095	3 889 777	2095	0.54 (0.51–0.56)	1.00 [Reference]
IDD diagnosis	20 684	20 430	42	2.06 (2.56–2.84)	3.82 (2.81–5.18)
Opioid use disorder					
No IDD diagnosis	3 931 095	17 197 944	24 041	1.40 (1.38–1.42)	1.00 [Reference]
IDD diagnosis	20 684	86 328	285	3.30 (2.94–3.71)	2.36 (2.10–2.65)
Dose escalation ≥90 MEQ					
No IDD diagnosis	3 931 095	123 074	48 561	394.6 (391.8–397.3)	1.00 [Reference]
IDD diagnosis	20 684	671	187	278.7 (246.7–314.7)	0.76 (0.66–0.88)
Propensity matched cohort					
Opioid toxicity					
No IDD diagnosis	76 708	76 250	107	1.40 (1.16–1.70)	1.00 [Reference]
IDD diagnosis	19 814	19 589	31	1.58 (1.11–2.25)	0.96 (0.63–1.47)
Opioid use disorder					
No IDD diagnosis	76 708	328 936	1028	3.13 (2.94–3.32)	1.00 [Reference]
IDD diagnosis	19 814	83 080	246	2.96 (2.61–3.35)	0.79 (0.68–0.91)
Dose escalation ≥90 MEQ					
No IDD diagnosis	76 708	2103	872	414.7 (394.2–436.3)	1.00 [Reference]
IDD diagnosis	19 814	612	170	277.6 (244.3–315.4)	0.80 (0.62–1.03)
High-dimensional propensity matched cohort					
Opioid toxicity					
No IDD diagnosis	72 007	71 550	87	1.22 (0.99–1.50)	1.00 [Reference]
IDD diagnosis	19 156	18 946	29	1.53 (1.06–2.20)	1.07 (0.70–1.65)
Opioid use disorder					
No IDD diagnosis	72 007	308 193	1023	3.32 (3.12–3.53)	1.00 [Reference]
IDD diagnosis	19 156	80 507	251	3.12 (2.76–3.53)	0.84 (0.73–0.97)
Dose escalation ≥90 MEQ					
No IDD diagnosis	72 007	1969	748	379.9 (359.0–401.9)	1.00 [Reference]
IDD diagnosis	19 156	592	170	286.9 (252.7–325.8)	0.92 (0.70–1.20)

IDD, intellectual and developmental disability; MEQ, milligrams of morphine or equivalent.

In the analysis of secondary outcomes, we found that there was a significantly higher hazard of new OUD diagnosis among those with IDD when compared with those without IDD in the unmatched analysis (3.30 *v*. 1.40 per 1000 person-years; hazard ratio 2.36, 95% CI 2.10–2.65). This association reversed after propensity score matching (hazard ratio 0.79, 95% CI 0.68–0.91) and HDPS matching (hazard ratio 0.84, 95% CI 0.73–0.97). In the unmatched analysis, IDD diagnosis was associated with a significantly lower hazard of dose escalation (279 *v*. 395 per 1000 person-years; hazard ratio 0.76, 95% CI 0.66–0.88) when compared with no IDD diagnosis (Table 3). This finding was also no longer significant after propensity score matching (hazard ratio 0.80, 95% CI 0.62–1.03) and HDPS matching (hazard ratio 0.92, 95% CI 0.70–1.20).

In our exploratory analysis examining correlates of opioid toxicity, we observed a number of variables that were significantly associated with a higher hazard of opioid toxicity, including younger age, male gender, rural residence, ADG score, number of emergency department visits, number of mental-health related physician visits, number of mental health hospital admissions, prior diagnosis of alcohol or other substance use disorders, prior stimulant or benzodiazepine use, higher daily dose of initial opioid prescription and having an initial prescription with a treatment duration of >7 days (Supplementary Table 6). In our on-treatment analysis, results were consistent with the primary analysis, although opioid toxicity events were rare (Supplementary Table 7).

## Discussion

In this large, population-based study of new opioid recipients, we found that initial prescription opioid characteristics were similar among individuals with and without IDD; however, an IDD diagnosis was associated with a higher hazard of opioid toxicity and OUD in the crude analysis. These risks were no longer present after accounting for differences in demographic and clinical characteristics between populations with and without IDD. This suggests that the IDD population is at a higher risk of opioid toxicity and OUD diagnosis after starting opioid therapy, and that this risk is not a result of the IDD diagnosis itself, but rather the clustering of known risk factors for opioid toxicity and substance use disorder that are often present in the IDD population.

Individuals with IDD are often young, male, have a high comorbidity burden and a high prevalence of mental health diagnoses and substance use disorders,^7,20^ all characteristics typically associated with an elevated risk of opioid toxicity and OUD, as demonstrated in our exploratory multivariable analysis and in previous literature.^28,29^ Although it is reassuring that a diagnosis of IDD is not the main contributor to an elevated risk of opioid toxicity, the clustering of risk factors for opioid-related adverse events in this population remains concerning for a number of reasons. First, inebriation among individuals with IDD can be difficult to identify because signs of intoxication can sometimes be overshadowed by characteristics of disability (e.g. decreased motor skills and slurred speech).^30^ Compounded by the fact that individuals with IDD continue to be infantilised,^31^ issues with substance use in this population are difficult to recognise until very obvious outcomes such as toxicity occur. This may partially explain our finding of a lower hazard of OUD diagnosis among those with IDD in our adjusted models as this change could reflect underdiagnosis of OUD in this population.

The results of our analyses present a unique opportunity to provide targeted care and education to the IDD population at the outset of opioid treatment, to support medication use while avoiding risks of opioid-related harm. Specifically, when considering opioid therapy for an individual with IDD, physicians should assess the patient's characteristics (demographics and medical history) in the context of known risk factors for opioid-related harm to avoid diagnostic overshadowing,^32^ and consider non-opioid alternatives when appropriate. When opioids are necessary, additional resources may be needed to help further patient's and/or caregiver's understanding of opioid-related risks and mitigation strategies.

### Strengths and limitations

This was a large, population-based study conducted with all adults in Ontario who started prescription opioid therapy, but there are several limitations that warrant further discussion. First, we relied on records of IDD-related encounters with the healthcare system to identify those with IDD. Therefore, individuals with IDD who did not access the healthcare system for disability-related morbidities would be misclassified in our cohort. Lunsky et al have demonstrated that the methods used to identify the IDD population in this study identify approximately two-thirds of all adults in Ontario with IDD diagnoses.^20^ However, those not included according to our definition of IDD likely had very mild IDD and accessed the healthcare system similarly to those without IDD. Therefore, this misclassification is unlikely to alter our results. Second, although we were unable to determine pain severity, the balance achieved on comorbidities and initial opioid characteristics after matching likely mitigated this difference. Third, we ascertained opioid exposure by using prescription dispensing records, and were therefore unable to determine whether an individual consumed the medication. However, this limitation likely applies to both exposure groups similarly. Fourth, we cannot differentiate toxicities from prescription opioids versus the unregulated drug supply, and therefore are unable to determine whether drug poisonings were related to dispensed medications. However, by restricting our cohort to individuals with no recent toxicity event or OUD diagnosis, it is possible that the outcomes captured in this study are reflective of prescription opioid-related harms or harms following transition from prescription opioids to the unregulated supply. Finally, our data do not include information on genetic markers that may contribute to different types of IDD diagnoses, and we were therefore unable to stratify our analyses by IDD type. This is an important direction for future research.

In conclusion, although characteristics of new opioid use are similar between individuals with and without IDD, those with IDD are at higher risk of experiencing opioid toxicity and being newly diagnosed with an OUD than those without IDD. These risks are driven by a high concentration of known risk factors for opioid-related harm in this population, but are no longer present after accounting for demographic and clinical population characteristics. Thus, clinicians considering opioid therapy for their patients should assess individuals with IDD similarly to those without, and should ensure that counselling on opioid risk mitigation approaches are developed specifically for those with IDD, to facilitate safe use of opioids to manage pain in this population.

## Data Availability

The data-sets from this study are held securely in coded form at ICES. Although data-sharing agreements prohibit ICES from making them publicly available, access may be granted to those who meet pre-specified criteria for confidential access, available at www.ices.on.ca/DAS. The full data-set creation plan and underlying analytic code are available from the corresponding author, Q.G., upon request, understanding that the computer programs may rely upon coding templates or macros that are unique to ICES and are therefore either inaccessible or may require modification.

## Supplementary material

For supplementary material accompanying this paper visit http://doi.org/10.1192/bjo.2022.612. click here to view supplementary material
