# Supplementary material for: New opioid use and risk of opioid-related adverse events among adults with intellectual and developmental disabilities in Ontario, Canada
Source: BJPsych Open. 2022 Nov 28;8(6):e208. doi: 10.1192/bjo.2022.612 (PMC9707500; doi:10.1192/bjo.2022.612)
Supplement: Supplementary file 1 [file S2056472422006123sup001.docx]

**SUPPLEMENTAL MATERIALS**

[Data sources](#_Toc98675832)

[e-Table 1. Palliative care and opioid toxicity diagnosis codes](#_Toc98675833)

[e-Table 2. Diagnosis codes related to developmental disabilities from the international classification of diseases (ICD), 9^th^ and 10^th^ editions, used to identify cohort of adults with IDD](#_Toc98675834)

[e-Table 3. Diagnostic criteria for codes in e-Table 2 to identify individuals with IDD](#_Toc98675835)

[high dimensional propensity score matching](#_Toc98675836)

[e-Table 4. Cohort characteristics stratified by IDD diagnosis, before and after high dimensional propensity score matching](#_Toc98675837)

[e-Table 5. Medication characteristics on index date and during observation window (365 days after opioid therapy initiation), before and after high dimensional propensity score matching](#_Toc98675838)

[e-Table 6. Correlates of opioid toxicity](#_Toc98675839)

[e-Table 7. Hazard of on-treatment opioid toxicity during the year after initiating opioid therapy. Results from the unmatched, PS-matched, and HDPS-matched analyses](#_Toc98675840)

# **Data Sources**

We used the Narcotics Monitoring System database, which holds records of all controlled substances dispensed in community pharmacies in Ontario (i.e., opioids, barbiturates, benzodiazepines, and stimulants), to obtain details of all prescription medications dispensed within our cohort, regardless of payer. We used the Canadian Institutes of Health Information (CIHI) Discharge Abstract Database (DAD) to identify patient-level information on inpatient hospitalizations, the CIHI-National Ambulatory Care Reporting System (NACRS) database to obtain information on emergency department visits, the CIHI-Same Day Surgery (SDS) database to capture day surgery information, and the Ontario Mental Health Reporting Systems (OMHRS) database to gather information on inpatient hospitalizations in psychiatric facilities. We captured information on outpatient physician visits using the Ontario Health Insurance Plan (OHIP) database and demographics as well as vital statistics using the Registered Persons Database. We identified instances of opioid-related deaths using the Drug and Drug/Alcohol Related Death database. We also used a collection of registries and validated databases from ICES to define additional patient comorbidities, including the Ontario Hypertension Dataset,^1^ the Chronic Obstructive Pulmonary Disease database,^2^ the Ontario Diabetes Dataset,^3^ Congestive Heart Failure database,^4^ the Ontario Rheumatoid Arthritis Dataset,^5,6^ the Ontario Myocardial Infarction Dataset,^7^ the Ontario HIV Database,^8^ and the Ontario Cancer Registry.

1. Tu K, Chen Z, Lipscombe LL, for the Canadian Hypertension Education Program Outcomes Research Taskforce. Prevalence and incidence of hypertension from 1995 to 2005: a population-based study. *Can Med Assoc J*. 2008;178(11):1429-1435. doi:10.1503/cmaj.071283

2. Gershon AS, Wang C, Guan J, Vasilevska-Ristovska J, Cicutto L, To T. Identifying Individuals with Physcian Diagnosed COPD in Health Administrative Databases. *COPD J Chronic Obstr Pulm Dis*. 2009;6(5):388-394. doi:10.1080/15412550903140865

3. Guttmann A, Nakhla M, Henderson M, et al. Validation of a health administrative data algorithm for assessing the epidemiology of diabetes in Canadian children. *Pediatr Diabetes*. 2010;11(2):122-128. doi:10.1111/j.1399-5448.2009.00539.x

4. Schultz SE, Rothwell DM, Chen Z, Tu K. Identifying cases of congestive heart failure from administrative data: a validation study using primary care patient records. *Chronic Dis Inj Can*. 2013;33(3):160-166.

5. Widdifield J, Bernatsky S, Paterson JM, et al. Accuracy of Canadian health administrative databases in identifying patients with rheumatoid arthritis: A validation study using the medical records of rheumatologists. *Arthritis Care Res*. Published online 2013:n/a-n/a. doi:10.1002/acr.22031

6. Widdifield J, Bombardier C, Bernatsky S, et al. An administrative data validation study of the accuracy of algorithms for identifying rheumatoid arthritis: the influence of the reference standard on algorithm performance. *BMC Musculoskelet Disord*. 2014;15(1):216. doi:10.1186/1471-2474-15-216

7. Austin PC, Daly PA, Tu JV. A multicenter study of the coding accuracy of hospital discharge administrative data for patients admitted to cardiac care units in Ontario. *Am Heart J*. 2002;144(2):290-296. doi:10.1067/mhj.2002.123839

8. Antoniou T, Zagorski B, Loutfy MR, Strike C, Glazier RH. Validation of Case-Finding Algorithms Derived from Administrative Data for Identifying Adults Living with Human Immunodeficiency Virus Infection. Thiem U, ed. *PLoS ONE*. 2011;6(6):e21748. doi:10.1371/journal.pone.0021748

| e-Table 1. Palliative care and opioid toxicity diagnosis codes | |
| --- | --- |
| **Database** | **Codes** |
| *Palliative care codes* | |
| Ontario Health Insurance Plan Database (OHIP) | A945, B998, C945, C882, C982, K023, W872, W882, W972, W982 |
| CIHI Discharge Abstract Database | PATSERV=58 |
| *Opioid toxicity codes* | |
| CIHI Discharge Abstract Database  CIHI National Ambulatory Care Reporting System | ICD-10: T40.0, T40.1, T40.2, T40.3, T40.6, |
| Drug and Drug/Alcohol Related Death database | Opioid-related death |

| **e-Table 2.** Diagnosis codes related to developmental disabilities from the International Classification of Diseases (ICD), 9^th^ and 10^th^ editions, used to identify cohort of adults with IDD | |
| --- | --- |
| **Code** | **Description** |
| *ICD-9* |  |
| 299 - 299.99 | Pervasive developmental disorders (eg. autism) |
| 317 - 317.99 | Mental retardation |
| 318 - 318.99 | Mental retardation |
| 319 - 319.99 | Mental retardation |
| 758.0 - 758.39 | Chromosomal anomalies for which a developmental disability is typically present |
| 758.5 | Other conditions due to autosomal anomalies |
| 758.8, 758.89 | Other conditions due to chromosome anomalies (do not include 758.81) |
| 758.9 | Conditions due to anomaly of unspecified chromosome |
| 759.5 | Tuberous sclerosis |
| 759.81 | Other and unspecified congenital anomalies: Prader-Willi syndrome |
| 759.821 | Other and unspecified congenital anomalies: de Lange syndrome (include only if 6 digits exist) |
| 759.827 | Other and unspecified congenital anomalies: Seckel syndrome (include only if 6 digits exist) |
| 759.828 | Other and unspecified congenital anomalies: Smith-Lemli-Opitz syndrome (include only if 6 digits exist) |
| 759.83 | Other and unspecified congenital anomalies: Fragile X syndrome |
| 759.874 | Other and unspecified congenital anomalies: Beckwith-Wiedemann syndrome (include only if 6 digits exist) |
| 759.875 | Other and unspecified congenital anomalies: Zellweger syndrome (include only if 6 digits exist) |
| 759.89 | Other and unspecified congenital anomalies: other (eg. Menkes disease, Laurence-Moon-Biedl syndrome, Rubinstein-Taybi syndrome) |
| 760.71 | Fetal alcohol syndrome |
| 760.77 | Fetal hydantoin syndrome |
| *ICD-10* |  |
| F700 | Mild mental retardation with the statement of no, or minimal, impairment of behaviour |
| F701 | Mild mental retardation, significant impairment of behaviour requiring attention or treatment |
| F708 | Mild mental retardation, other impairments of behaviour |
| F709 | Mild mental retardation without mention of impairment of behaviour |
| F710 | Moderate mental retardation with the statement of no, or minimal, impairment of behaviour |
| F711 | Moderate mental retardation, significant impairment of behaviour requiring attention or treatment |
| F718 | Moderate mental retardation, other impairments of behaviour |
| F719 | Moderate mental retardation without mention of impairment of behaviour |
| F720 | Severe mental retardation with the statement of no, or minimal, impairment of behaviour |
| F721 | Severe mental retardation, significant impairment of behaviour requiring attention or treatment |
| F728 | Severe mental retardation, other impairments of behaviour |
| F729 | Severe mental retardation without mention of impairment of behaviour |
| F730 | Profound mental retardation with the statement of no, or minimal, impairment of behaviour |
| F731 | Profound mental retardation, significant impairment of behaviour requiring attention or treatment |
| F738 | Profound mental retardation, other impairments of behaviour |
| F739 | Profound mental retardation without mention of impairment of behaviour |
| F780 | Other mental retardation with the statement of no, or minimal, impairment of behaviour |
| F781 | Other mental retardation, significant impairment of behaviour requiring attention or treatment |
| F788 | Other mental retardation, other impairments of behaviour |
| F789 | Other mental retardation without mention of impairment of behaviour |
| F790 | Unspecified mental retardation with the statement of no, or minimal, impairment of behaviour |
| F791 | Unspecified mental retardation, significant impairment of behaviour requiring attention or treatment |
| F798 | Unspecified mental retardation, other impairments of behaviour |
| F799 | Unspecified mental retardation without mention of impairment of behaviour |
| F840 | Childhood autism |
| F841 | Atypical autism |
| F843 | Other childhood disintegrative disorder |
| F844 | Overactive disorder associated with mental retardation and stereotyped movements |
| F845 | Asperger’s syndrome |
| F848 | Other pervasive developmental disorders |
| F849 | Pervasive developmental disorders, unspecified |
| Q851 | Tuberous sclerosis |
| Q860 | Fetal alcohol syndrome |
| Q861 | Fetal hydantoin syndrome |
| Q871 | Aarskog, Prader-Willi, de Lange, Seckel, etc. |
| Q8723 | Rubinstein-Taybi syndrome (include only if all 5 digits exist) |
| Q8731 | Sotos syndrome (include only if all 5 digits exist) |
| Q878 | Other |
| Q900 - Q939 except Q926 | All Down syndrome types, cri du chat, etc. (except extra marker chromosomes) |
| Q971 | Female with more than three X chromosomes |
| Q992 | Fragile x syndrome |
| Q998 | Other specified chromosome abnormalities |

| **e-Table 3.** Diagnostic criteria for codes in e-Table 2 to identify individuals with IDD | | | |
| --- | --- | --- | --- |
| **Database** | **Year of Inception** | **Diagnoses** | **Criteria** |
| Discharge Abstract Database | 1988 | Discharges with any diagnosis listed in **Table 1** (above) | -any diagnostic field  -for all facilities submitting to DAD, SDS and NACRS  -from inception database to just before accrual period (December 31, 2012) |
| Same Day Surgery Database | 1991 |  |  |
| National Ambulatory Care Reporting System | 2002 |  |  |
| Ontario Mental Health Reporting System | 2005 | Q3=1  **or** Q2a_retired_2016, Q2b_retired_2016 or Q2c_retired_2016 is 299 to 299.80 before fiscal year 2016; Q2a_retired_2019, Q2b_retired_2019 or Q2c_retired_2019 is 299 to 299.80 between fiscal year 2016 and fiscal year 2018 inclusive  **or** Q2d_retired_2016 is 317 to 319.99 for before fiscal year 2016; Q2d_retired_2019 is 317 to 319.99 between fiscal year 2016 and fiscal year 2018 inclusive  **or** I11h-I11m = any one of the following ICD-10 codes: Q851, Q860, Q861, Q871, Q8723, Q8731, Q878, Q900-Q939 (but not Q926), Q971, Q992, Q998 | -for all faculties submitting to OMHRS  -from inception of database to just before accrual period (December 31, 2012) |
| Ontario Health Insurance Plan | 1991 | 299, 319 | -for all providers submitting to OHIP  -from June 1991 to right before accrual period (December 2012) |

# **High Dimensional Propensity Score Matching**

This method balances exposure groups through the use of large health administrative datasets in order to capture unmeasured confounders by proxy.^1^ Using the HDPS algorithm available at ICES, we captured the following data dimensions during the six months prior to index for all individuals in the cohort: all medication drug claims for controlled substances (1 dimension), all hospitalization and emergency department diagnoses and procedures (4 dimensions), and all physician procedures and diagnoses in the outpatient setting (2 dimensions). The algorithm gathered the top 200 most prevalent covariates from each dimension and converted each into three binary variables based on how often the covariates recurred (once, sporadic, frequent). The covariates were then prioritized using a multiplicative bias based on the prevalence of the covariate among the IDD and general population group, as well as the association between covariate and the outcome. The top 500 covariates with the highest bias terms were used to estimate the high-dimensional propensity score, along with age, sex, income quintile and rurality. Once we calculated the HDPS estimate for each individual in the cohort, we matched the IDD group to the general population group using the same criteria listed above for the propensity score matching.

Reference:

1. Austin, P. C., Wu, C. F., Lee, D. S. & Tu, J. V. Comparing the high-dimensional propensity score for use with administrative data with propensity scores derived from high-quality clinical data. *Stat. Methods Med. Res.* **29**, 568–588 (2020).

| **e-Table 4.** Cohort characteristics stratified by IDD diagnosis, before and after high dimensional propensity score matching | | | | | | | | |
| --- | --- | --- | --- | --- | --- | --- | --- | --- |
|  | **Unmatched Cohort** | |  | **High Dimensional Propensity Score Matched Cohort** | | | |  |
|  | **IDD** | **No IDD** |  | **IDD** | | | **No IDD** | |
|  | **N=20,684** | **N=3,931,095** |  | **N=19,156** | | | **N=72,007** | |
| **Demographics** | | | |  |  | | | |
| Age, mean ± SD | 37.2 ± 17.0 | 49.7 ± 18.7 ^1^ |  | 36.9 ± 17.0 | | | 36.9 ± 17.0 | |
| Male, No. (%) | 11,943 (57.7) | 1,829,924 (46.5) ^1^ |  | 11,054 (57.7) | | | 41,548 (57.7) | |
| Income Quintile, No. (%) |  |  |  |  | | |  | |
| 1 | 6,053 (29.3) | 764,644 (19.5) ^1^ |  | 5,533 (28.8) | | | 20,594 (28.6) | |
| 2 | 4,457 (21.5) | 784,699 (20.0) |  | 4,142 (21.6) | | | 15,121 (21.0) | |
| 3 | 3,592 (17.4) | 789,542 (20.1) |  | 3,378 (17.6) | | | 12,961 (18.0) | |
| 4 | 3,328 (16.1) | 796,431 (20.3) ^1^ |  | 3,094 (16.2) | | | 11,953 (16.6) | |
| 5 | 3,225 (15.6) | 790,458 (20.1) ^1^ |  | 3,009 (15.7) | | | 11,449 (15.9) | |
| Rural Residence | 2,549 (12.3) | 437,599 (11.1) |  | 2,363 (12.3) | | | 9,073 (12.6) | |
| **Health Services Utilization, mean ± SD** | | | | | |  |  |  |
| Number of ED Visits | 1.7 ± 4.2 | 0.7 ± 1.3 ^1^ |  | 1.4 ± 2.9 | | | 1.2 ± 2.2 | |
| Number of Physician Office Visits (any reason) | 7.9 ± 8.4 | 7.2 ± 7.0 |  | 7.5 ± 7.9 | | | 7.4 ± 8.0 | |
| Number of Physician Office Visits (mental health) | 1.8 ± 4.8 | 0.5 ± 2.4 ^1^ |  | 1.6 ± 4.2 | | | 1.4 ± 4.2 | |
| Previous Hospitalization (N, %) | 2,467 (11.9) | 304,221 (7.7) ^1^ |  | 2,015 (10.5) | | | 6,985 (9.7) | |
| Previous Mental Health Hospitalization (N, %) | 1,222 (5.9) | 12,925 (0.3) ^1^ |  | 934 (4.9) | | | 1,656 (2.3) ^1^ | |
| **Comorbidities and Medication Use, No. (%)** | | | | | |  |  |  |
| ADG, mean ± SD | 6.7 ± 3.9 | 6.0 ± 3.4^1^ |  | 6.4 ± 3.8 | | | 6.3 ± 3.6 | |
| Alcohol and Substance Use Disorder | 712 (3.4) | 51,256 (1.3) ^1^ |  | 602 (3.8) | | | 2,592 (3.6) | |
| Cancer | 1,028 (5.0) | 353,406 (9.0) ^1^ |  | 955 (5.0) | | | 3,672 (5.1) | |
| COPD | 1,649 (8.0) | 384,844 (9.8) |  | 1,468 (7.7) | | | 4,536 (6.3) | |
| Diabetes | 2,628 (12.7) | 559,363 (14.2) |  | 2,395 (12.5) | | | 7,129 (9.9) | |
| Hypertension | 3,828 (18.5) | 1,255,805 (31.9) ^1^ |  | 3,527 (18.4) | | | 12,529 (17.4) | |
| Rheumatoid Arthritis | 146 (0.7) | 53,192 (1.4) |  | 132 (0.7) | | | 576 (0.8) | |
| Benzodiazepine Use (6 months prior to index) | 4,377 (21.2) | 299,370 (7.6) ^1^ |  | 3,433 (17.9) | | | 11,377 (15.8) | |
| Stimulant Use (6 months prior to index) | 1,161 (5.6) | 28,130 (0.7) ^1^ |  | 999 (5.2) | | | 3,168 (4.4) | |

*^1^ Meaningful difference based on standardized difference >0.10 when compared to IDD group*

*Abbreviations: ADG, Aggregated Diagnosis Group; COPD, chronic obstructive pulmonary disease; ED, emergency department; IDD, intellectual and developmental disability; SD, standard deviation*

| **e-Table 5.** Medication characteristics on index date and during observation window (365 days after opioid therapy initiation), before and after high dimensional propensity score matching | | | | | | | | |
| --- | --- | --- | --- | --- | --- | --- | --- | --- |
|  | **Unmatched Cohort** | |  | **High Dimensional Propensity Score Matched Cohort** | | | |  |
|  | **IDD** | **No IDD** |  | **IDD** | | | **No IDD** | |
|  | **N=20,684** | **N=3,931,095** |  | **N=19,156** | | | **N=72,007** | |
| **Index Opioid Characteristics, No. (%)** | | | |  |  | | | |
| Single Opioid Prescription on Index | 20,479 (99.0) | 3,891,330 (99.0) |  | 19,017 (99.3) | | | 71,503 (99.3) | |
| Opioid Type |  |  |  |  | | |  | |
| Codeine | 495 (2.4) | 70,861 (1.8) |  | 445 (2.3) | | | 1,152 (1.6) | |
| Codeine Combination | 13,143 (63.5) | 2,265,336 (57.6) ^1^ |  | 12,320 (64.3) | | | 43,708 (60.7) | |
| Fentanyl | ≤5 | 516 (0) |  | ≤5 | | | ≤5 | |
| Hydromorphone | 1,594 (7.7) | 313,666 (8.0) |  | 1,371 (7.2) | | | 4,824 (6.7) | |
| Meperidine | 46 (0.2) | 9,605 (0.2) |  | 46 (0.2) | | | 216 (0.3) | |
| Methadone (for pain) | 0 | ≤5 |  | 0 | | | 0 | |
| Morphine | 760 (3.7) | 112,757 (2.9) |  | 680 (3.6) | | | 2,016 (2.8) | |
| Oxycodone | 237 (1.1) | 80,473 (2.0) |  | 209 (1.1) | | | 1,080 (1.5) | |
| Oxycodone Combination | 3,211 (15.5) | 723,388 (18.4) |  | 2,958 (15.4) | | | 13,177 (18.3) | |
| Other Opioids | 1,304 (6.3) | 374,355 (9.5) ^1^ |  | 1,213 (6.3) | | | 6,193 (8.6) | |
| Long-Acting Opioid | 160 (0.8) | 35,074 (0.9) |  | 53 (0.3) | | | 216 (0.3) | |
| Daily Dose > 50 MEQ | 3,131 (15.1) | 654,164 (16.6) |  | 2,790 (14.6) | | | 9,937 (13.8) | |
| Days’ Supply > 7 Days | 3,184 (15.4) | 796,832 (20.3) ^1^ |  | 2,871 (15.0) | | | 11,593 (16.1) | |
| Concurrent Benzodiazepine Prescription | 3,166 (15.3) | 204,046 (5.2) ^1^ |  | 2,443 (12.8) | | | 7,129 (9.9) | |
| Concurrent Stimulant Prescription | 899 (4.3) | 20,358 (0.5) ^1^ |  | 762 (4.0) | | | 2,304 (3.2) | |
| **Observation Window Medication Characteristics, No (%)** | | | | | |  |  |  |
| Additional Opioid Prescription Dispensed | 6,043 (29.2) | 1,264,823 (32.2) |  | 5,501 (28.7) | | | 21,890 (30.4) | |
| Long-Acting Opioid during observation window | 388 (1.9) | 93,112 (2.4) |  | 308 (1.6) | | | 1,152(1.6) | |
| Number of additional opioid prescriptions, mean ± SD | 1.2 ± 5.7 | 0.9 ± 3.1 |  | 1.1 ± 4.5 | | | 0.9 ± 3.4 | |
| Opioid Discontinuation | 20,462 (98.9) | 3,901,431 (99.2) |  | 18,975 (99.1) | | | 71,575 (99.4) | |

*^1^ Meaningful difference based on standardized difference >0.10 when compared to IDD group*

*Abbreviations: IDD, intellectual and developmental disability; MEQ, milligrams of morphine or equivalent; SD, standard deviation*

*Abbreviations: CI, confidence interval; IDD, intellectual and developmental disability; MEQ, milligrams of morphine or equivalent*

| **e-Table 6.** Correlates of Opioid Toxicity | | | | |
| --- | --- | --- | --- | --- |
|  | **Hazard Ratio** | **95% Confidence Interval** | **P-Value** |  |
| IDD diagnosis | 1.45 | 1.06 – 2.00 | 0.02 |  |
| Age: 18-24 years | 2.56 | 2.21 – 2.98 | <0.0001 |  |
| Age: 25-34 years | 1.87 | 1.62 – 2.16 | <0.0001 |  |
| Age: 35-44 years | 1.18 | 1.01 – 1.38 | 0.04 |  |
| Age: 45-54 years | 1.19 | 1.03 – 1.37 | 0.02 |  |
| Age: 55-64 years | 0.80 | 0.68 – 0.93 | 0.01 |  |
| Male | 1.23 | 1.13 – 1.34 | <0.0001 |  |
| Rural residence | 1.22 | 1.08 – 1.39 | 0.01 |  |
| Total ADG score | 1.12 | 1.10 – 1.14 | <0.0001 |  |
| Number of hospitalizations | 1.08 | 1.01 – 1.14 | 0.02 |  |
| Number of emergency department visits | 1.03 | 1.02 – 1.03 | <0.0001 |  |
| Number of mental health office visits | 1.06 | 1.05 – 1.07 | <0.0001 |  |
| Number of mental health hospitalizations | 1.09 | 1.04 – 1.14 | 0.0002 |  |
| Number of office visits (any reason) | 0.97 | 0.96 – 0.98 | <0.0001 |  |
| Alcohol or substance use disorder diagnosis | 6.05 | 5.28 – 6.94 | <0.0001 |  |
| Overlapping stimulant use | 1.99 | 1.49 – 2.67 | <0.0001 |  |
| Overlapping benzodiazepine use | 2.86 | 2.53 – 3.22 | <0.0001 |  |
| Daily dose of initial opioid | 1.01 | 1.00 – 1.01 | 0.0001 |  |
| Initial opioid days’ supply >7 days | 1.71 | 1.53 – 1.92 | <0.0001 |  |
| Initial opioid daily dose >50 MEQ | 1.00 | 0.84 – 1.19 | 0.99 |  |

| **e-Table 7.** Hazard of on-treatment opioid toxicity during the year after initiating opioid therapy. Results from the unmatched, PS-matched, and HDPS-matched analyses | | | | | | |
| --- | --- | --- | --- | --- | --- | --- |
|  | **Total Number of Individuals** | | **Total Number of Person-Years of Follow-Up** | **Number of Individuals with Outcome** | **Incidence Rate per 1,000 Person-Years (95% CI)** | **Hazard Ratio (95% CI)** |
| **Unmatched** | | | | | | |
| No IDD Diagnosis | 3,931,095 | 129,764 | | 524 | 4.04 (3.71 - 4.40) | 1.00 [reference] |
| IDD Diagnosis | 20,684 | 703 | | 9 | 12.8 (6.69 - 24.5) | 3.11 (1.61 – 6.01) |
| **PS Matched** | | | | | | |
| No IDD Diagnosis | 76,708 | | 2,205 | 17 | 7.71 (4.80 - 12.38) | 1.00 [reference] |
| IDD Diagnosis | 19,814 | | 641 | 7 | 12.2 (6.15 – 24.39) | 1.34 (0.40 - 4.46) |
| **HDPS Matched** | | | | | | |
| No IDD Diagnosis | 72,007 | | 2,063 | 16 | 7.75 (4.76 - 12.63) | 1.00 [reference] |
| IDD Diagnosis | 19,156 | | 620 | 7 | 11.29 (5.40 - 23.58) | 2.00 (0.51 - 7.85) |

*Abbreviations: CI, confidence interval; HDPS, high-dimensional propensity score; IDD, intellectual and developmental disability; PS, propensity score*
